# Mutations in the Homeodomain of HOXD13 Cause Syndactyly Type 1-c in Two Chinese Families

**DOI:** 10.1371/journal.pone.0096192

**Published:** 2014-05-01

**Authors:** Limeng Dai, Dan Liu, Min Song, Xueqing Xu, Gang Xiong, Kang Yang, Kun Zhang, Hui Meng, Hong Guo, Yun Bai

**Affiliations:** 1 Department of Medical Genetics, College of Basic Medical Sciences, Third Military Medical University, Chongqing, China; 2 Department of Thoracic and Cardiac Surgery, Southwest Hospital, Third Military Medical University, Chongqing, China; National Eye Institute, United States of America

## Abstract

**Background:**

Syndactyly type 1 (SD1) is an autosomal dominant limb malformation characterized in its classical form by complete or partial webbing between the third and fourth fingers and/or the second and third toes. Its four subtypes (a, b, c, and d) are defined based on variable phenotypes, but the responsible gene is yet to be identified. SD1-a has been mapped to chromosome 3p21.31 and SD1-b to 2q34–q36. SD1-c and SD1-d are very rare and, to our knowledge, no gene loci have been identified.

**Methods and Results:**

In two Chinese families with SD1-c, linkage and haplotype analyses mapped the disease locus to 2q31-2q32. Copy number variation (CNV) analysis, using array-based comparative genomic hybridization (array CGH), excluded the possibility of microdeletion or microduplication. Sequence analyses of related syndactyly genes in this region identified c.917G>A (p.R306Q) in the homeodomain of *HOXD13* in family A. Analysis on family B identified the mutation c.916C>G (p.R306G) and therefore confirmed the genetic homogeneity. Luciferase assays indicated that these two mutations affected the transcriptional activation ability of *HOXD13*. The spectrum of *HOXD13* mutations suggested a close genotype-phenotype correlation between the different types of *HOXD13*-Syndactyly. Overlaps of the various phenotypes were found both among and within families carrying the *HOXD13* mutation.

**Conclusions:**

Mutations (p.R306Q and p.R306G) in the homeodomain of *HOXD13* cause SD1-c. There are affinities between SD1-c and synpolydactyly. Different limb malformations due to distinct classes of *HOXD13* mutations should be considered as a continuum of phenotypes and further classification of syndactyly should be done based on phenotype and genotype.

## Introduction

Syndactyly (SD) is a digital malformation in which adjacent fingers and/or toes are webbed because they fail to separate during limb development. It is one of the most common hereditary limb malformations with a prevalence of 3–10 in 10 000 births, though higher estimates ranging from 10–40/10 000 have been reported [1]–[2]. The current classification scheme defines nine types syndactyly on the basis of phenotype and genotype, which is helpful in the understanding of syndactyly malformation and in defining its affinity with other digit anomalies, including polydactyly, oligodactyly, and brachydactyly [3]. Of all the known non-syndromic syndactylies, SD1 is one of the most common types and follows an autosomal dominant inheritance pattern with variable phenotypes. Webbing may affect fingers and/or toes, be unilateral or bilateral, cutaneous or bony, and reach the level of the nail or solely affect the proximal segments of the digits. Based on clinical observations and current classification, SD1 can be further divided into four subtypes (a, b, c, and d).

SD1-a (Weidenreich type; zygodactyly; OMIM 609815) was mapped to chromosome 3p21.31 and characterized by bilateral webbing of 2/3 toes without hand anomalies. The features of SD1-b (Lueken type; OMIM 185900) are bilateral cutaneous 3/4 fingers and webbing of 2/3 toes. It was mapped to chromosome 2q34-q36 and the chromosome locus was designated as the SD1 locus [4]–[5]. SD1-c, also named Montagu type or 3/4 fingers syndactyly, is characterized by unilateral or bilateral cutaneous or bony webbing of 3/4 fingers and occasionally of 4/5 fingers. It is recognized that the feet are not involved in this subtype [6]–[7]. SD1-d (Castilla type) has only been observed in an epidemiological study [2] and is characterized by bilateral cutaneous webbing of 4/5 toes. To date, little is known about these four subtypes and the gene locus for the rare SD1-c and SD1-d subtypes has not been identified.

*HOX* genes encode a set of highly conserved transcription factors that control cell fate and the regional identities along the primary body and limb axes in metazoans [8]. There are 39 *HOX* genes arranged in four separate clusters (*HOXA–D*) in mammalian genomes. HOX proteins bind specific DNA sequences via their homeodomains, and are thought to regulate overlapping sets of target genes, although the molecular pathways controlled by HOX proteins are only starting to be unveiled [9]. *HOXD13*, the gene at the 5′ end of the HOXD cluster, is the first *HOX* gene known to be linked to human developmental disorders. It has two coding exons: exon 1 with the imperfect GCN (where N denotes A, C, G, or T) triplet repeats encoding the N-terminal region with a 15-residue polyalanine tract, and exon 2, which contains the homeobox region encoding the C-terminal portion with a 60-residue homeodomain [10]. Mutations in the human *HOXD13* gene could lead to diversiform phenotypes, including synpolydactyly type 1 (SPD1; OMIM 186000), brachydactyly types D (BDD; OMIM 113200) and E (BDE; OMIM 113300), SD5 (OMIM 186300), a novel brachydactyly-syndactyly syndrome (BD-SD; OMIM 610713), or VACTERL association (OMIM 192350).

In this study, our aim was to further characterize the SD1-c phenotype based on two Chinese families with 3/4 fingers syndactyly. We discovered that two missense mutations in codon 306 in the homeodomain of *HOXD13* underlie SD1-c.

## Materials and Methods

### Ethics Statement

The study was approved by the Third Military Medical University institutional review board. A written informed consent was obtained from all participants and/or guardians on the behalf of the minors/children participants.

### Families

**Figure 1:**
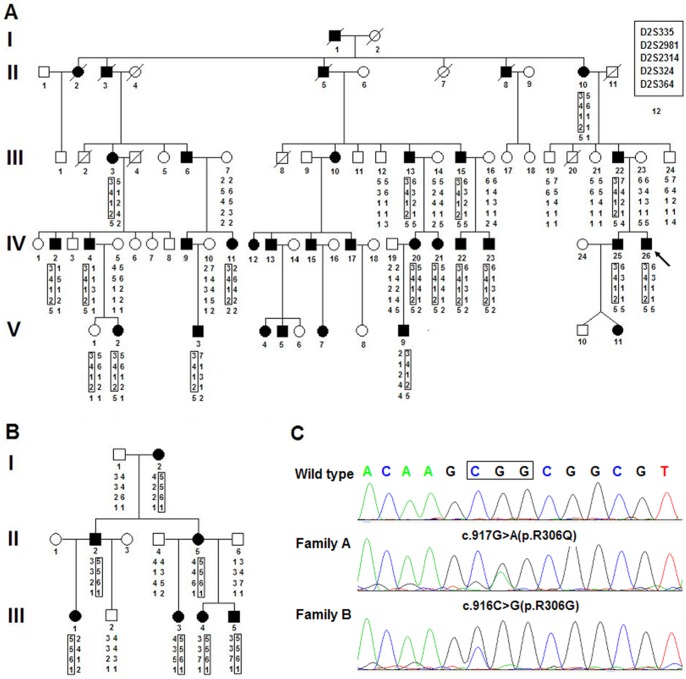
Pedigree, haplotype analysis, and mutation analysis of the families in this study. **A:** Pedigree and disease-haplotype segregation of family A. Blackened symbols represent affected individuals with an abnormal limb phenotype. White symbols represent individuals with a normal limb phenotype. Circles and squares indicate females and males, respectively. The arrows identify the proband and the disease-haplotype is boxed. **B:** Pedigree and disease-haplotype segregation of family B. **C:** *HOXD13* missense mutations in the two families.

Two Chinese Han families (Figure 1A and 1B) were ascertained in the present study. After physical examination, digital photographs and radiographs were taken to document the limb phenotypes in some of the individuals.

**Figure 2:**
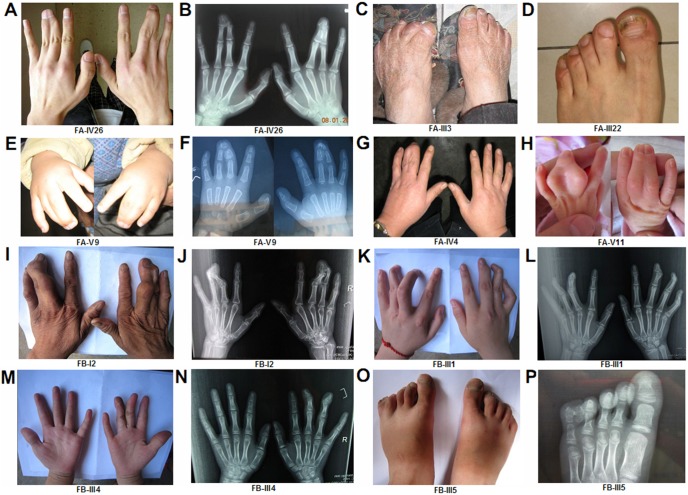
Photographs and radiographs of individuals. Phenotypic spectrum of the two families showing typical SD1-c bilateral 3/4 fingers syndactyly (**A, B, H, I and J**) and unilateral 3/4 fingers syndactyly (**G, M and N**); transformative SD1-c bilateral 3/4 finger syndactyly with right fifth finger clinodactyly (**K and L**); mild SPD, bilateral 3/4 fingers syndactyly with phalanx duplication (**E and F**) and unilateral 3/4 fingers syndactyly with 4/5 fingers synpolydactyly (**O and P**). Other phenotypes included isolated unilateral partial webbing of 2/3 toes like SD1-a (**D**) and isolated unilateral adduction deformity of toes (**C**).

Family A (FA) consisted of 74 individuals including 33 affected individuals (12 females and 21 males) of five generations. The proband (FA-IV26) had complete bilateral webbing of 3/4 fingers, where the webbing included a fusion of nails according the phenotype and radiographs. His feet were normal and no other abnormalities were noted. The phenotypes varied from partial unilateral to complete bilateral cutaneous webbing of 3/4 fingers in the other affected individuals. Among the 18 affected individuals available for phenotypic evaluation, 13 had the same phenotype as the proband, 2 had unilateral cutaneous webbing of 3/4 fingers (FA-IV4, FA-V2) and only FA-V9 had a mild SPD phenotype of the hands with normal feet. Notably, two affected individuals (FA-III3, FA-III22) presented with an atypical abnormality of the feet but without hand anomalies. FA-III3 had a unilateral adduction deformity of the toes. FA-III22 had unilateral partial cutaneous webbing of 2/3 proximal toes (Figure 1A, Figure 2, Figure S1 and Table 1).

**Table 1:** The clinical spectrum in family A and family B with SD1-c.

Subject ID	Phenotype	Webbing in fingers		Webbing in toes		Additional finding	
		Right hand	Left hand	Right foot	Left foot		
FA-II10;III6,III10,III13,III15;IV2,IV9,IV11,IV13,IV15,IV17,IV20,IV21,IV23,IV25,IV26;V2,V3,V4,V5,V11							
	SD1-c	3/4 fingers	3/4 fingers	/	/	**/**	
FA-IV4	SD1-c	/	3/4 fingers	/	/	**/**	
FA-V2	SD1-c	3/4 fingers	/	/	/	**/**	
FA-V9	Mild SPD1	/	3/4 fingers	/	/	bilateral phalanx duplication of hands	
FA-III3	SD1-a	/		/	2/3 proximal toes; partial cutaneous	/	
FA-III22	unknown	/	/	/	/	adduction deformity of left toes	
FB-I2;II2,II5; III3	SD1-c	3/4 fingers	3/4 fingers	/	/	/	
FB-III1	SD1-c	3/4 fingers	3/4 fingers		/	5th finger clinodactyly of right hand	
FB-III4	SD1-c	3/4 fingers	/	/	/	/	
FB-III5	Mild SPD			/	4/5 toes	unilateral phalanx duplication of left foot	

Family B (FB) consisted of 13 members, including 7 affected individuals (5 females and 2 males). All members were available for clinical evaluations. Five affected individuals had a complete bilateral webbing of 3/4 fingers with normal feet. One of them (FB-III1) also had clinodactyly of the fifth finger. In addition, FB-III4 presented unilateral cutaneous webbing of 2/3 fingers with normal feet. Notably, FB-III5 had a unilateral synpolydactyly of 4/5 toes and unilateral cutaneous webbing of 3/4 fingers (Figure 1B, Figure 2, Figure S1 and Table 1).

### Linkage and Haplotype Analyses

As we known, linkage analysis remains a very good choice for large family and could clearly reflect the disease locus. Short tandem repeat (STR) typing was performed firstly in present families. Blood samples were collected and genomic DNA was extracted from 29 and 11 members of family A and family B, respectively. We carried out a search of the whole chromosome 2 (30 microsatellite markers) and chromosome 3p21.31-3p22.31 region (2 microsatellite markers) on the base of Linkage Mapping Set v2.5. Three additional microsatellites (D2S2981, D2S2314, and D2S324) were selected using the UCSC Genome Browser on Human Mar. 2009 Assembly (http://www.genome.ucsc.edu) to further refine the critical region of the disease locus. Two-point LOD scores were calculated using the MLINK program of the FASTLINK package, assuming that the disease in the family is inherited in an autosomal dominant mode with penetrance of 0.95, the disease-allele frequency is 0.0003, and allele frequencies are equal at all the marker loci.

### Microarray-based Comparative Genomic Hybridization

Array-based comparative genomic hybridization (array CGH) was carried out using the Roche NimbleGen Genome-Wide array CGH 3*720K containing over 720,000 copy-number probes. Genomic DNA samples were genotyped at the CapitalBio Corporation (Beijing, China) with the CGH array in accordance with the manufacturer’s protocols. Genotype calling, genotyping quality control, and copy number variation (CNV) identification were performed with the Roche NimbleGen SignalMap software. The data accessed at Gene Expression Omnibus (GEO, No: GSE55181).

### Mutation Screening

Exons and splice sites of syndactyly-related genes *HOXD13, GJA1* and zone of polarizing activity regulatory sequence (*ZRS*) were amplified by polymerase chain reaction (PCR) using gene-specific primers. Amplified fragments were sequenced using an ABI 3130 automated sequencer (Applied Biosystems, Foster City, CA, USA).

### Review of the *HOXD13* Mutations

The name of *HOXD13* mutations in related studies is confusing because it is uncertain whether Met-1 or Met-9 is the initiator at present. The primitive sequence AAC51635.1 contains 335 amino acids and the newly accession NP_000514.2 contains 343 amino acids, which could result from erroneous and uncertain initiation. Thus, we reviewed the *HOXD13* mutations based on sequence NP_000514.2 to better understand its mutation spectrum.

### Functional Significance Prediction

Three online programs: PolyPhen2 (http://genetics.bwh.harvard.edu/pph2), SIFT (http://sift.jcvi.org), and Swiss-Model (http://swissmodel.expasy.org), were used to predict the functional significance of mutations.

### Plasmid Construction and Luciferase Assays

The human *EPHA7* gene promoter of 660 bp (from −580 to +80), which contains a HOXD13 binding site, was obtained by PCR from human genomic DNA and insert into the XhoI and HindIII sites of a pGL3-basic vector (Promega, Madison, WI, USA) to generate a pGL3-EPHA7 reporter construct. Human HOXD13 of pReceiver-M02 expression clone was obtained from GeneCopoeia (Rockville, MD, USA). Site-directed mutagenesis was performed with appropriate primers to generate HOXD13 carrying the c.916C>G (p.R306G), c.917G>A (p.R306Q) or c.964A>C (p.I322L) mutations using the QuickChange Lightning Site-DirectedMutagenesis kit (Strata-gene, La Jolla, CA, USA). Presence of the desired base changes was verified by DNA sequencing.

The 293FT cells were cultured in 1640 supplemented with 10% FBS, and seeded in 24-well tissue culture plates 24 h prior to transfection at about 80% confluence. Plasmids were transfected with lipofectamine2000 (Invitrogen) according to the manufacturer’s instructions. HOXD13 expression vectors or the control vectors were cotransfected with pGL3 reporter vectors along with pRL-TK vectors (Renilla luciferase, Promega) using lipofectamine2000. Cell was collected 24 h after transfection and luciferase activities were measured using Dual-Luciferase Reporter Assay System (promega). Activity was defined as Firefly/Renilla ratio.

## Results

### Segregation Analysis

In family A, the syndactyly did not skip any generations, indicating that the disease is of autosomal dominant inheritance. Segregation analysis of the pedigree confirmed this mode of inheritance. Results from the two-point linkage analysis provided strong evidence that the SD1-c disease locus in this family linked to STR markers of chromosome 2q31-2q32. The maximum LOD score calculated was 4.88 at a recombination fraction of 0.00 and penetrance of 0.95 at the D2S335 locus. The LOD scores of 2q33.3-q36.3 and 3p21.31-3p22.1 regions were insufficient to support the linkage relationship. Haplotype analysis demonstrated that all of the 17 affected members examined had a common haplotype of 3-4-1-2 for four STR marker loci: D2S335, D2S2981, D2S2314, and D2S324. Based on haplotype analysis, recombination events were observed in FA-IV11 and FA-V1. One unaffected individual (FA-V1) carried a common disease-associated haplotype and the sequencing results demonstrated that the individual carried a mutation. (Figure 1A and Table 2).

**Table 2:** Two-point LOD score of chromosome 2q markers at various recombination fractions.

Position		Marker	LOD score at theta (penetrance = 0.95)				
			0.0	0.1	0.2	0.3	0.4
**2q31.1-2q31.3**							
		D2S335	4.88	4.12	3.15	2.03	0.86
		D2S2981	4.13	3.15	2.72	1.81	0.81
		D2S2314	3.15	2.68	2.08	1.41	0.69
		D2S324	4.65	3.92	3.00	1.95	0.83
		D2S364	0.87	3.24	2.61	1.77	0.81
**2q33.3-q36.3**							
		D2S325	–1.97	0.88	1.11	0.86	0.41
		D2S2382	–2.99	0.26	0.29	0.20	0.09
		D2S126	–7.41	–0.75	–0.16	0.03	0.05
		D2S396	–13.51	–1.86	–0.82	–0.37	–0.13
**3p21.31-3p22.1**							
		D3S1581	–3.56	–0.35	–0.19	–0.12	–0.07
		D3S3685	–3.82	–0.09	–0.84	–0.33	–0.09

In family B, the common region was also confirmed. A haplotype of 5-5-6-1 of this four STR marker loci (D2S335, D2S2981, D2S2314 and D2S324) was found in all patients. The maximum LOD score in family B was 1.79. (Figure1B and Table 2).

### CNV Analysis

To determine whether the SD1-c phenotype was caused by an unknown microdeletion or microduplication, we performed a genome-wide high-resolution CNV scan of an affected individual (male, FA-IV4) and a healthy control (male FA-IV8). However, we did not detect any potential pathogenic CNVs in the critical regions (GSE55181), including the *ZRS* region which is the cause of SD4.

### Mutation Analysis

Direct sequencing of *HOXD13* revealed a heterozygous G-to-A transition in exon 2 at position 917 of the coding sequence in the proband of family A. This base change converted amino acid 306 from arginine to glutamine. The same base change was identified by direct sequencing in other affected family members and one unaffected individual (FA-V1). In the proband of family B, there was a G-to-C transversion at position 916, thus leading to the change of amino acid 306 from arginine to glycine. These two missense mutations were not found in unaffected family members except FA-V1 or in the 100 unaffected healthy control subjects (Figure 1C). No mutations were found in the *GJA1* gene and *ZRS* region.

### A Spectrum of *HOXD13* Mutations

**Figure 3:**
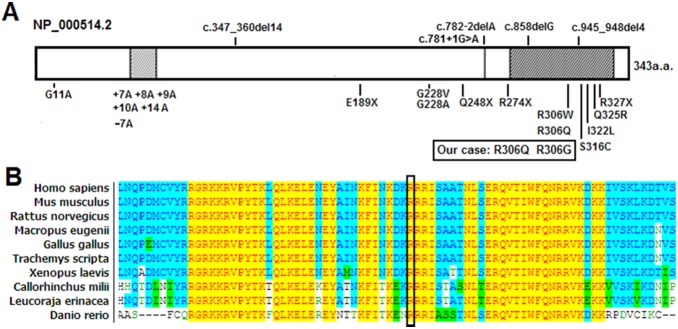
Schematic presentation of the HOXD13 structure and sequences alignment analysis. **A:** Schematic presentation of the HOXD13 structure and annotated mutations identified in families. **B:** Partial amino acid sequences alignment in homeodomain of HOXD13 among several species. The position of mutated amino acid of our cases is indicated by the black box.

Our results provide new information and enlarge the spectrum of known *HOXD13* mutations. To date, more than 20 different variants of the *HOXD13* gene have been reported from different families [9], [11]–[28]. Some mutations with unspecified phenotypes have also been found in sporadic cases [29]. The present cases in combination with those studied previously suggest that mutations in the polyalanine tract and homeodomain are in the majority of families with *HOXD13* mutations. These data suggest that mutations in these regions of the protein underlie the majority of *HOXD13*-associated cases and that this region may be particularly important for protein function (Figure 3A and Table 3).

**Table 3:** Overview of annotated HOXD13 mutations and clinical observations in family members or sporadic cases.

Phenotype	Source	Report based onAAC51635.1	Report based on NP_000514.2	Combined data based on NP_000514.2	Type of mutation	Clinical observations in affected family members or sporadic cases	Reference
SD1-c	Family		c.C916G;p.R306G	c.C916G;p.R306G	missense	3/4 fingers SD; 3/4 fingers SD with 5th finger clinodactyly; 3/4 fingers SD and 4/5 toes SD with a phalanx duplication between 4/5 toes	Our case
SD1-c	Family		c.G917A;p.R306Q	c.G917A;p.R306Q	missense	3/4 fingers SD; 3/4 fingers SD with a phalanx duplication; partial cutaneous 2/3 proximal toes; other deformities	Our case
SPD	Family		c.G32C;p.G11A	c.G32C;p.G11A	missense	bilateral clinodactyly of the 5th finger with discrete shortening and external rotation of 4th and 5th toes (heterozygous); complex SPD with clinical overlap with brachydactyly type A1 and B (homozygous)	[12]
SPD	Family	+7A +8A +9A +10A +14A		+7A +8A +9A +10A +14A	Ala expansion	3/4 fingers SPD with 4/5 toes SPD; 3/4 fingers SD; 3/4 fingers SD with 4/5 toes SPD; 2/3 fingers SD and 5/6 toes SD; slight deformities without fingers SD with 5/6 SD; 3/4/5/6 fingers SD; incomplete formation of the limb, digits and brachydactyly	[13]–[17]
SPD	Family	c.323–336del14		c.347_360del14	frameshift	3/4 fingers SPD; 4/5 toes SPD	[18]
SPD	Family	c.G659T;p.G220V		c.G683T;p.G228V	missense	3/4 fingers SPD; 5th finger clinodactyly; partial cutaneous SD of 2/3 toes and 3/4 toes	[9]
SPD	Family	c.G659C;p.G220A		c.G683T;p.G228A	missense	3/4 fingers SPD; 5th finger clinodactyly	[30]
SPD	Family	c.C718T;p.Q240X		c.C742T;p.Q248X	nonsense	3/4/5 toes SPD; 3/4/5 SD; ectrodactyly and SD; 3/4 toes SD; cleft lip	[19]
SPD	Family		c.C742T;p.Q248X	c.C742T;p.Q248X	nonsense	3/4 fingers SPD; SD involving toes 2/3/4/5 or 3/4/5; camptodactyly; big toe; brachydactyly; 2/3 toes SD; 4/5 toes SD	[20]
SPD	Family		c.781+1G>A;p.G190fsX4	c.781+1G>A;p.G190fsX4	splicing;frameshift	3/4 fingers SD; 3/4 fingers SPD; 4/5 toes SD; 5th toes polydactyly; hallux clinodactyly	[31]
SPD	Family	c.834delG		c.858delG	frameshift	3/4 fingers SPD; 4/5 toes SPD	[18]
SPD	Family	c.C892T;p.R298W		c.916T;p.R306W	missense	3/4 fingers SPD; 5th finger clinodactyly	[21]
SPD	Family	c.C955T;p.R319X		c.979T;p.R327X	nonsense	5th fingers clinodactyly	[22]
atypical SD	Family	c.758–2delA		c.782–2delA	splicing;frameshift	3/4 fingers SD; 5th fingers clinodactyly; symphalangism of toes	[23]
atypical SD	Family	c.G893A;p.R298Q		c.G917A; p.R306Q	missense	3/4 fingers SD; 2nd finger clinodactyly	[11]
SD5	Family	c.A950G;p.Q317R		c.A974G;p.Q325R	missense	4/5 metacarpal synostosis; fusion extended to the phalanges of 4/5 fingers; shortening or clinodactyly of the 5th fingers; 3/4 fingers SD; 2/3 toes SD; 3/4 toes SD; 4/5 SPD	[24]
BD-SD	Family	-7A		-7A	Ala contraction	distal phalanges of the thumbs; 2/3 toes SD; short proximal phalanges of toes; shortened middle phalanges fused with the distal ones	[24]
BDE	Family		c.C820T;p.R274X	c.C820T;p.R274X	nonsense	shortened 5th fingers and toes	[26]
BDE/BDD	Family	c.C923G;p.S308C		c.C947G;p.S316C	missense	thumb distal phalangeal brachydactyly; metacarpal brachydactyly; long or short distal digit phalanges	[27]
BDE/BDD	Family	c.A940C;p.I314L		c.A964C;p.I322L	missense	little finger distal phalangeal hypoplasia/absence; 3th metacarpal brachydactyly/ring finger lateral duplication; 3/4 fingers SD	[27]
Not-specified	sporadic	-19 relative to AUG		uncertain	uncertain	thumb polydactyly	[29]
Not specified	sporadic	c.24delC		c.48delC	frameshift	4/5 toes SPD	[29]
Not specified	sporadic	-4A		–4A	Ala contraction	4/5 toes SPD; thumb polydactyly	[29]
Not specified	sporadic	c.G8C;p.G3A		c.G32C;p.G11A	missense	right thumb polydactyly; right 3/4 toes SD	[29]
Not specified	sporadic	c.C43A;p.P15T		c.C67A;p.P23T	missense	left thumb polydactyly	[29]
Not specified	sporadic	c.C97G,p.R33G		c.C121G;p.R41G	missense	right thumb polydactyly	[29]
Not specified	sporadic	c.133_139insGGGC		c.157_163insGGGC	insertion	4/5 toes SPD	[29]
Not specified	sporadic	c.G143A;p.R48Q		c.G167A;p.R104Q	missense	left 5/6 toes SPD	[29]
Not specified	Family	c.G541T;p.E181X		c.G565T;p.E189X	nonsense	not specified	[25]
Not specified	Family	c.921_924del4		c.945_948del4	frameshift	not specified	[26]
VACTERL	sporadic	-7A		–7A	Ala contraction	fusion of the distal inter-phalangeal joints of the 4th and 5th toes	[28]

### Functional Analysis

The p.R306Q and p.R306G mutations altered the amino acids at residue 31 of the homeodomain. An alignment of HOXD13 protein sequences showed that this position is highly conserved among many different species (Figure 3B). Thus, this amino acid appears to play an important role in the structure and function of the HOXD13 protein.

The R306Q and R306G substitutions were rated as ‘probably damaging’ by PolyPhen2 analysis, with a score of 1.00 (sensitivity: 0.00; specificity: 1.00). Other misssense mutations were also rated as ‘probably damaging’. However, Swiss-Model analysis indicated that the overall structures of the mutant proteins did not differ substantially from the wild-type protein.

**Figure 4:**
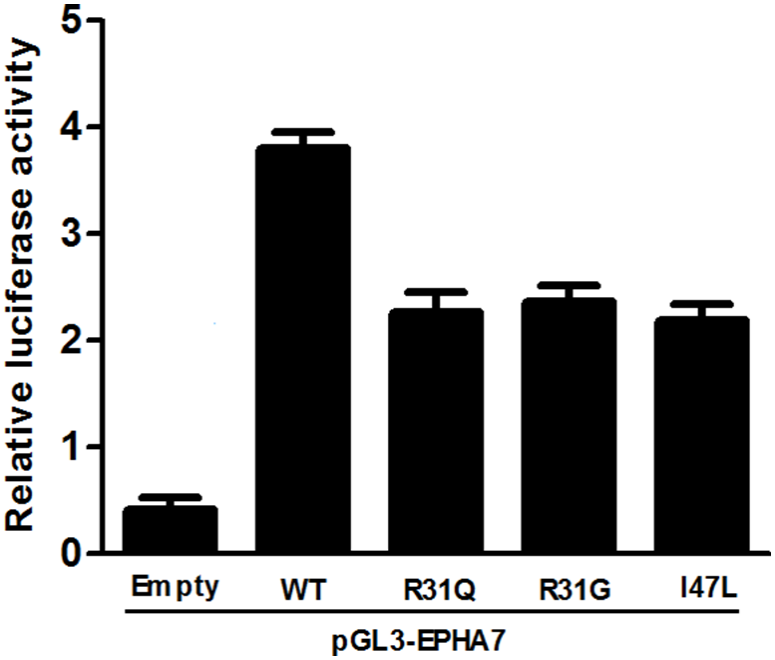
Transcriptional activity of wild-type and mutant HOXD13 proteins at the human *EPH7A* promoter. The R31Q (p.R306Q), R31G (p.R306G) and I47L (p.I322L) mutants show a significantly diminished stimulation compared with the wild-type control at approximately 62%, 60% and 58% of the wild-type HOXD13, respectively. Bars represent firefly/Renilla luciferase ratios for the different constructs.

Luciferase assays were conducted to determine whether the mutation affected the capability of HOXD13 protein to activate transcription. The pGL3-EPHA7 luciferase reporter constructs were tested and the c.964A>C (p.I322L) mutant that converts residue 47 of the homeodomain from isoleucine to leucine was also examined (approx. 58% of wild type). The c.916C>G (p.R306G) and c.917G>A (p.R306Q) mutants also showed significantly diminished stimulation compared with the wild-type control (approx. 62% and 60% of wild-type, respectively). Thus, these two mutations affected the capacity of HOXD13 to activate transcription (Figure 4).

## Discussion

SD1-c was first described by Motagu in 1953 and is characterized by unilateral or bilateral, cutaneous or bony fusion of 3/4 fingers with normal feet [7]. Another large Chinese family with SD1-c, reported by Hsu, had 23 affected subjects demonstrating variable degrees of bilateral osseous fusion of 3/4 or 3/4/5 fingers. Only one subject had partial fusion of the toes from the third to the fifth [6]. The disease followed an autosomal dominant inheritance pattern with phenotypic heterogeneity and incomplete penetrance in the families of the present study. Most affected individuals exhibited isolated bilateral syndactyly of 3/4 fingers, while a small number were unilateral (FA-IV4, FA-V2, FB-III4). Other particular phenotypes were also observed as follows: (1) unilateral abnormal foot without hand anomalies; FA-III3 only presented with unilateral adduction malformation of the toes and FA-III22 had a unilateral partial webbing of 2/3 toes overlapping the typical appearance of SD1-a. (2) Occasional mild SPD phenotype; FA-V9 had bilateral SPD (3/4 fingers) without foot anomalies; FB-III5 had unilateral 3/4 fingers syndactyly with a mild unilateral phenotype of SPD (4/5 toes). (3) A rare individual with forme fruste (FA-V1) was observed. The families in our study confirmed the genetic homogeneity of previous SD1-c studies and the typical SD1-c phenotype was observed in almost all affected individuals with only a small number of affected individuals exhibiting an alternative appearance.

There were some common associations between SD1 and SPD. The typical feature of SD1-c is isolated syndactyly of 3/4 fingers, while the cardinal features of SPD are the webbing of 3/4 fingers and 4/5 toes, with partial or complete digital duplication within the syndactylous web. The webbing phenotype (3/4 fingers) was recognized as a milder SPD phenotype in several SPD families. In addition, the SD1-a phenotype (isolated webbing of 2/3 toes) was occasionally found in SPD families [32] [33]. The close association between SD1-c and SPD inspired us to determine the disease-associated gene for SD1-c. The evidence provided by linkage and haplotype analyses revealed that affected members of family A had a common haplotype of 3–4-1–2 for four STR marker loci, while family B had 5–5-6–1. The different haplotypes suggested that these two families were derived from different ancestors; therefore, the region was localized to 2q31-2q32. The evidence from overlapping phenotypic and genetic analyses indicated that abnormalities in *HOXD13* may be the common cause of SD1-c and SPD. Finally, the result of the direct sequencing of *HOXD13* confirmed our hypothesis. Two *HOXD13* mutations, p.R306Q and p.R306G, were identified in the families of the present study, respectively. At the same time, CNV analysis using array CGH excluded the cause of an unknown microdeletion or microduplication, such as in the *ZRS* region. In our previous study of SD4, one individual that carried a *ZRS* duplication exhibited a postaxial polydactyly of the right foot similar to SPD [34].

The *HOXD13* gene is associated with SD-1c in the presented families. Other limb malformations associated with mutations in *HOXD13* included SPD1, BDD, BDE, SD5, BD-SD syndrome, and VACTERL. They provide a large dataset for analysis of the most common mutations and of the different types of mutations found, thus generating a preliminary genotype-phenotype correlation. Our present report and the observations of others are an indication of the existence of genotype-phenotype correlation for the limb morphopathies caused by *HOXD13* mutations, especially in the homeodomain.

The homeodomains of the *HOX* genes are DNA-binding motifs composed of a flexible N-terminal region followed by three α-helices and are highly conserved among different species of the HOXD13 protein. In previous studies, five missense mutations have been identified in the homeodomain (R298W, R298Q, S308C, I314L and Q317R based on AAC51635.1, also known as residues R31W, R31Q, S41C, I47L and Q50R of the homeodomain), each producing distinctive phenotypes. Interestingly, the two mutations (R306Q and R306G based on NP_000514.2, also named R31W and R31G) located in the same codon (306) of present two families also exhibit a different phenotype from that reported previously.

The R31W mutation was previously in one Danish family reported by Debeer et al. Fifteen affected individuals had bilateral fifth finger clinodactyly. Three of those also had mild unilateral SPD, which was radiologically documented in two cases as cutaneous syndactyly of 3/4 fingers, with a duplicated distal phalanx in the syndactylous web. In addition, one subject was clinically unaffected but an obligate mutation carrier [21]. The R31Q mutation was documented in a Chinese family by Wang et al. In that study, three individuals presented with an isolated bilateral cutaneous webbing of 3/4 fingers. Three had bilateral clinodactyly of the index fingers and one had bilateral clinodactyly of the second toe. In the family A of our present study, the R31Q mutation was observed, where twenty-three affected individuals exhibited isolated bilateral or unilateral 3/4 fingers syndactyly. Three affected individuals exhibited appearance of mild SPD1, SD1-a or isolated unilateral toe adduction. The R31G mutation was observed in family B and six affected individuals had isolated bilateral or unilateral 3/4 syndactyly, one affected individual had unilateral 3/4 fingers syndactyly with unilateral mild phenotype of SPD. Moreover, carriers of the S41C and I47L mutations displayed a unique brachydactyly-polydactyly syndrome, overlapping with BDE, with considerable variations in severity and expressivity. Carriers of Q50R displayed another special phenotype of SD5.

This phenotypic distinction might arise from the specific impact of different amino acids on protein stability or its affinity for binding DNA recognition elements. An R31P missense mutation in the homeodomain of human MSX1 led to partial or complete loss of function because of a perturbation of the structure and diminished ability compared with the wild-type gene [35]. *EPHA7* gene is downstream of HOXD13 and acts during limb development. HOXD13 can bind to a single evolutionarily conserved site within the *EPHA7* promoter and activates its transcription [36]. Transactivation assays also demonstrated that some mutations affected the transcriptional activation ability of HOXD13. Both the R31Q and I47L mutants showed a significantly reduced transcription activation ability of the *EPHA7* promoter compared with the wild type [11]. The Q50R mutation resulted in a more reduced activation than the I47L mutation [24]. The proximal cartilages in embryos with ectopic expression of HOXD13 (IQN), an artificial mutant carrying alanine substitutions at positions 47(I), 50(Q) and 51(N) of the homeodomain, which renders it unable to bind DNA, were shorter than in the embryos with expression of wild-type HOXD13 [37]. Our Luciferase assays demonstrated that R31 and G31 mutants also changed the DNA-binding ability. All missense mutations in HOXD13 were rated as ‘probably damaging’ by PolyPhen2 analysis. This suggests that mutations in the homeodomain result in a partial loss of function in the transcription-regulating functions of HOXD13. Therefore, an alteration of arginine would affect the ability of HOXD13 to make the salt bridges normally formed by R31. This phenotypic distinction might arise from the impact of different amino acid residues on the stability of the protein structure or the DNA-binding ability of the homeodomain to the DNA recognition elements. Alternatively, some unidentified genetic factors, except the impact of CNV, might correlate with *HOXD13* mutations resulting in varied phenotypes. Therefore, the substitution would affect the structure and lead to loss of function.

We re-examined the proposed genotype-phenotype correlation for HOXD13 (Figure 3 and Table 3). The polyalanine repeat expansions and frameshift deletion mutations usually give rise to typical SPD phenotypes, and the homeodomain missense mutation cluster for brachydactyly types [38]. Close genotype-phenotype correlations were also observed between different types of *HOXD13*-Syndactyly. Overlap of various phenotypes could be found among and within families carrying *HOXD13* mutation. The same minor malformations may be associated with different clinical abnormalities of the limb. For example, soft-tissue syndactyly of 2/3 toes were observed in families with mild SPD, BD-SD syndrome, SD5 [24], SD1-a, SD1-b or SD1-c. Additionally, cutaneous syndactyly of 3/4 fingers without digit duplication was observed in mild SPD, SD5, SD1-b or SD1-c. In the same codon of the same gene, the phenotype of R31 mutations in HOXD13 exhibited an interesting continuous phenotype: from unilateral to bilateral, from SD1-c phenotype to isolated 3/4 finger SPD and typical SPD (3/4 finger SPD and 4/5 toe SPD), from mild SPD to severe SPD and other phenotypes. No definite line could be drawn between the milder SPD variants and the SD1 phenotypes. Therefore, different limb malformations due to distinct classes of HOXD13 mutations should be considered as a continuum of phenotypes. “HOXD13 limb morphopathies” appears to be the most appropriate term to cover all limb malformations due to HOXD13 mutations in accordance with Zhao et al., Malik et al., and Brions et al. [24]–[25], [38].

Typing of SD1-c may be quite puzzling since phenotypic heterogeneity may lead to a clinical misclassification. Intra-familial and individual-specific clinical variability in SD1-c families may exhibit phenotypic overlap with other entities such as SD1-a, SPD or atypical limb malformations (e.g. clinodactyly). The same phenotype could be caused by different gene mutations and mutations in the same gene could also lead to different phenotypes such as that of the *HOXD13* mutation. In addition, different missense mutations in the same codon exhibited phenotypic heterogeneity. Individuals with rare forme fruste further reflect the complexity of phenotype. The heterogeneity may partly reflect individual lifestyles and specific types of mutation. In addition, environmental factors and other genetic factors such as genetic polymorphisms linked to SD-associated genes and epigenetic regulation of the expression of these genes may be involved in these syndromes. Whole exome sequencing of these families could provide important insights in the future. At present, more weight should be given to the phenotypes seen in large families for the correct typing of SD (and other developmental anomalies) and the most common phenotype in these large families should be considered firstly for typing [3].

In conclusion, mutations (p.R306Q and p.R306G) in the homeodomain of *HOXD13* could cause SD1-c. There are affinitis between the SD-1c and SPD phenotypes. Our study provides new information regarding the involvement of *HOXD13* gene mutations in the clinical and genetic diversity of SD, and advances our understanding of human limb development. Different limb malformations due to the distinct classes of *HOXD13* mutation should be considered as a continuum of phenotypes and further classification of syndactyly should be done based on phenotype and genotype. The term “HOXD13 limb morphopathies” seems appropriate to cover all limb malformations due to *HOXD13* mutations.

## Supporting Information

Figure S1 Photographs of affected individuals in family A and family B.
(PDF)

## References

[1] Hay S (1971) Incidence of selected congenital malformations in Iowa. Am J Epidemiol 94: 572–584. 4257973 10.1093/oxfordjournals.aje.a121356

[2] Castilla EE, Paz JE, Orioli-Parreiras IM (1980) Syndactyly: frequency of specific types. Am J Med Genet 5: 357–364. 6249121 10.1002/ajmg.1320050406

[3] Malik S (2012) Syndactyly: phenotypes, genetics and current classification. Eur J Hum Genet 20: 817–824. 22333904 10.1038/ejhg.2012.14 PMC3400728

[4] Bosse K, Betz RC, Lee YA, Wienker TF, Reis A, et al (2000) Localization of a gene for syndactyly type 1 to chromosome 2q34-q36. Am J Hum Genet 67: 492–497. 10877983 10.1086/303028 PMC1287194

[5] Ghadami M, Majidzadeh AK, Haerian BS, Damavandi E, Yamada K, et al (2001) Confirmation of genetic homogeneity of syndactyly type 1 in an Iranian family. Am J Med Genet 104: 147–151. 11746046 10.1002/ajmg.10061

[6] Hsu CK (1965) Hereditary syndactylia in a Chinese family. Chin Med J 84: 482–485. 5865199

[7] Montagu MF (1953) A pedigree of syndactylism of the middle and ring fingers. Am J Hum Genet 5: 70–72. 13050611 PMC1716452

[8] Favier B, Dolle P (1997) Developmental functions of mammalian Hox genes. Mol Hum Reprod 3: 115–131. 9239717 10.1093/molehr/3.2.115

[9] Fantini S, Vaccari G, Brison N, Debeer P, Tylzanowski P, et al (2009) A G220V substitution within the N-terminal transcription regulating domain of HOXD13 causes a variant synpolydactyly phenotype. Hum Mol Genet 18: 847–860. 19060004 10.1093/hmg/ddn410

[10] Akarsu AN, Stoilov I, Yilmaz E, Sayli BS, Sarfarazi M (1996) Genomic structure of HOXD13 gene: a nine polyalanine duplication causes synpolydactyly in two unrelated families. Hum Mol Genet 5: 945–952. 8817328 10.1093/hmg/5.7.945

[11] Wang B, Xu B, Cheng Z, Zhou X, Wang J, et al (2012) A novel non-synonymous mutation in the homeodomain of HOXD13 causes synpolydactyly in a Chinese family. Clin Chim Acta 413: 1049–1052. 22374128 10.1016/j.cca.2012.02.015

[12] Brison N, Debeer P, Fantini S, Oley C, Zappavigna V, et al (2012) An N-terminal G11A mutation in HOXD13 causes synpolydactyly and interferes with Gli3R function during limb pre-patterning. Hum Mol Genet 21: 2464–2475. 22373878 10.1093/hmg/dds060

[13] Muragaki Y, Mundlos S, Upton J, Olsen BR (1996) Altered growth and branching patterns in synpolydactyly caused by mutations in HOXD13. Science 272: 548–551. 8614804 10.1126/science.272.5261.548

[14] Wajid M, Ishii Y, Kurban M, Dua-Awereh MB, Shimomura Y, et al (2009) Polyalanine repeat expansion mutations in the HOXD13 gene in Pakistani families with synpolydactyly. Clin Genet 76: 300–302. 19686284 10.1111/j.1399-0004.2009.01213.x

[15] Malik S, Girisha KM, Wajid M, Roy AK, Phadke SR, et al (2007) Synpolydactyly and HOXD13 polyalanine repeat: addition of 2 alanine residues is without clinical consequences. BMC Med Genet 8: 78. 18072967 10.1186/1471-2350-8-78 PMC2222244

[16] Xin Q, Li L, Li J, Qiu R, Guo C, et al (2012) Eight-alanine duplication in homeobox D13 in a Chinese family with synpolydactyly. Gene 499: 48–51. 22406499 10.1016/j.gene.2012.02.046

[17] Gong L, Wang B, Wang J, Yu H, Ma X, et al (2011) Polyalanine repeat expansion mutation of the HOXD13 gene in a Chinese family with unusual clinical manifestations of synpolydactyly. Eur J Med Genet 54: 108–111. 20974300 10.1016/j.ejmg.2010.10.007

[18] Goodman F, Giovannucci-Uzielli ML, Hall C, Reardon W, Winter R, et al (1998) Deletions in HOXD13 segregate with an identical, novel foot malformation in two unrelated families. Am J Hum Genet 63: 992–1000. 9758628 10.1086/302070 PMC1377502

[19] Low KJ, Nwbury-Ecob RA (2012) Homozygous nonsense mutation in HOXD13 underlies synpolydactyly with a cleft. Clin Dysmorphol 21: 141–143. 22473151 10.1097/MCD.0b013e32835306f0

[20] Kurban M, Wajid M, Petukhova L, Shimomura Y, Christiano AM (2011) A nonsense mutation in the HOXD13 gene underlies synpolydactyly with incomplete penetrance. J Hum Genet 56: 701–706. 21814222 10.1038/jhg.2011.84 PMC4296310

[21] Debeer P, Bacchelli C, Scambler PJ, De Smet L, Fryns JP, et al (2002) Severe digital abnormalities in a patient heterozygous for both a novel missense mutation in HOXD13 and a polyalanine tract expansion in HOXA13. J Med Genet 39: 852–856. 12414828 10.1136/jmg.39.11.852 PMC1735011

[22] Furniss D, Kan SH, Taylor IB, Johnson D, Critchley PS, et al (2009) Genetic screening of 202 individuals with congenital limb malformations and requiring reconstructive surgery. J Med Genet 46: 730–735. 19429598 10.1136/jmg.2009.066027 PMC2764122

[23] Kan SH, Johnson D, Giele H, Wilkie AO (2003) An acceptor splice site mutation in HOXD13 results in variable hand, but consistent foot malformations. Am J Med Genet A 121A: 69–74. 12900906 10.1002/ajmg.a.20103

[24] Zhao X, Sun M, Zhao J, Leyva JA, Zhu H, et al (2007) Mutations in HOXD13 underlie syndactyly type V and a novel brachydactyly-syndactyly syndrome. Am J Hum Genet 80: 361–371. 17236141 10.1086/511387 PMC1785357

[25] Brison N, Tylzanowski P, Debeer P (2012) Limb skeletal malformations - what the HOX is going on? Eur J Med Genet 55: 1–7. 21782042 10.1016/j.ejmg.2011.06.003

[26] Jamsheer A, Sowinska A, Kaczmarek L, Latos-Bielenska A (2012) Isolated brachydactyly type E caused by a HOXD13 nonsense mutation: a case report. BMC Med Genet 13: 4. 22233338 10.1186/1471-2350-13-4 PMC3278352

[27] Johnson D, Kan SH, Oldridge M, Trembath RC, Roche P, et al (2003) Missense mutations in the homeodomain of HOXD13 are associated with brachydactyly types D and E. Am J Hum Genet. 72: 984–997. 10.1086/374721 PMC1180360 12649808

[28] Garcia-Barcelo MM, Wong KK, Lui VC, Yuan ZW, So MT, et al (2008) Identification of a HOXD13 mutation in a VACTERL patient. Am J Med Genet A 146A: 3181–3185. 19006232 10.1002/ajmg.a.32426

[29] Nakano K, Sakai N, Yamazaki Y, Watanabe H, Yamada N, et al (2007) Novel mutations of the HOXD13 gene in hand and foot malformations. Int Surg 92: 287–295. 18399101

[30] Zhou X, Zheng C, He B, Zhu Z, Li P, et al (2013) A novel mutation outside homeodomain of HOXD13 causes synpolydactyly in a Chinese family. Bone 57: 237–241. 23948678 10.1016/j.bone.2013.07.039

[31] Shi X, Ji C, Cao L, Wu Y, Shang Y, et al (2013) A splice donor site mutation in HOXD13 underlies synpolydactyly with cortical bone thinning. Gene 532: 297–301. 24055421 10.1016/j.gene.2013.09.040

[32] Goodman FR, Mundlos S, Muragaki Y, Donnai D, Giovannucci-Uzielli ML, et al (1997) Synpolydactyly phenotypes correlate with size of expansions in HOXD13 polyalanine tract. Proc Natl Acad Sci U S A 94: 7458–7463. 9207113 10.1073/pnas.94.14.7458 PMC23843

[33] Sayli BS, Akarsu AN, Sayli U, Akhan O, Ceylaner S, et al (1995) A large Turkish kindred with syndactyly type II (synpolydactyly). 1. Field investigation, clinical and pedigree data. J Med Genet 32: 421–434. 7666393 10.1136/jmg.32.6.421 PMC1050481

[34] Dai L, Guo H, Meng H, Zhang K, Hu H, et al (2013) Confirmation of genetic homogeneity of syndactyly type IV and triphalangeal thumb-polysyndactyly syndrome in a Chinese family and review of the literature. Eur J Pediatr 172: 1467–1473. 23793141 10.1007/s00431-013-2071-y

[35] Hu G, Vastardis H, Bendall AJ, Wang Z, Logan M, et al (1998) Haploinsufficiency of MSX1: a mechanism for selective tooth agenesis. Mol Cell Biol 18: 6044–6051. 9742121 10.1128/mcb.18.10.6044 PMC109190

[36] Salsi V, Zappavigna V (2006) Hoxd13 and Hoxa13 directly control the expression of the EphA7 Ephrin tyrosine kinase receptor in developing limbs. J Biol Chem 281: 1992–1999. 16314414 10.1074/jbc.M510900200

[37] Caronia G, Goodman FR, McKeown CM, Scambler PJ, Zappavigna V (2003) An I47L substitution in the HOXD13 homeodomain causes a novel human limb malformation by producing a selective loss of function. Development 130: 1701–1712. 12620993 10.1242/dev.00396

[38] Malik S, Grzeschik KH (2008) Synpolydactyly: clinical and molecular advances. Clin Genet 73: 113–120. 18177473 10.1111/j.1399-0004.2007.00935.x

