# Correction: Mutations in the Homeodomain of HOXD13 Cause Syndactyly Type 1-c in Two Chinese Families

**DOI:** 10.1371/journal.pone.0104309

**Published:** 2014-07-28

**Authors:** The PLOS ONE Staff

The affiliation for the tenth author is incorrect. Yun Bai is not affiliated with #2 but with #1 Department of Medical Genetics, College of Basic Medical Sciences, Third Military Medical University, Chongqing, China.
