# Supplementary material for: Mutations in the Homeodomain of HOXD13 Cause Syndactyly Type 1-c in Two Chinese Families
Source: PLoS One. 2014 May 1;9(5):e96192. doi: 10.1371/journal.pone.0096192 (PMC4006867; doi:10.1371/journal.pone.0096192)

FA-II10

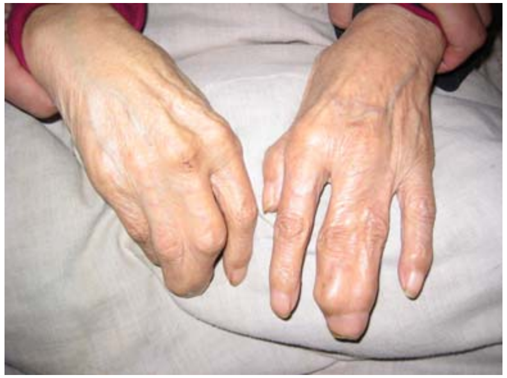

FA-III3

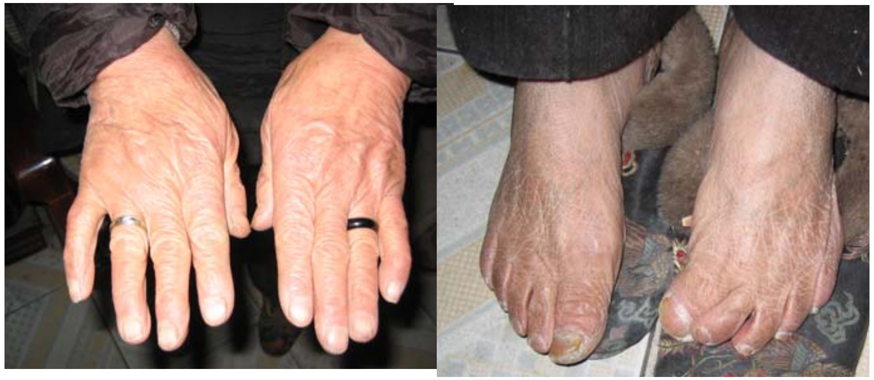

FA-III13

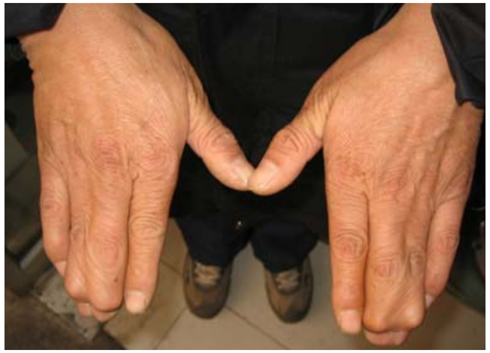

FA-III15

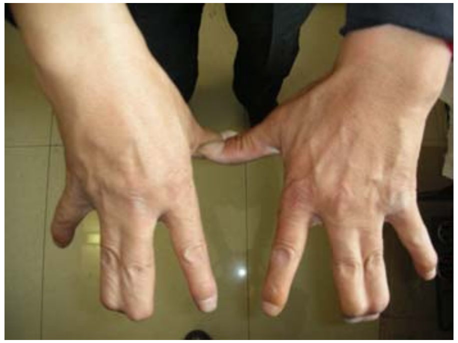

FA-III22

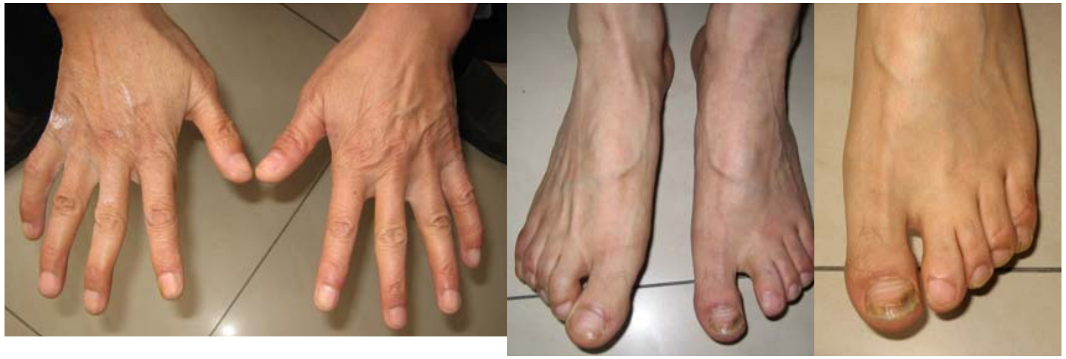

FA-IV2-surgery

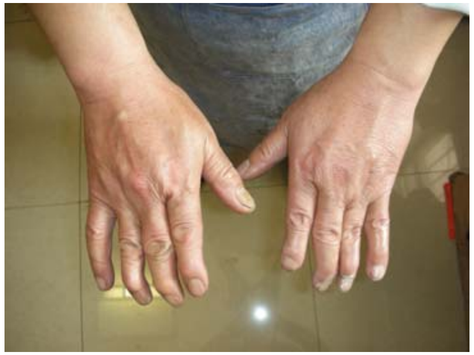

FA-IV4

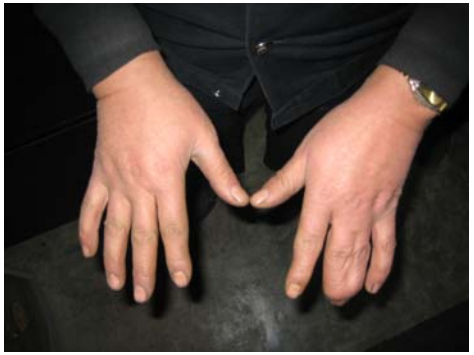

FA-IV11-surgery

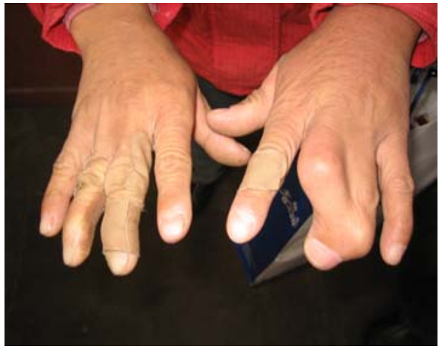

FA-IV20

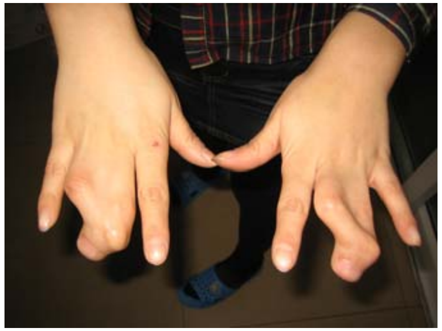

FA-IV21

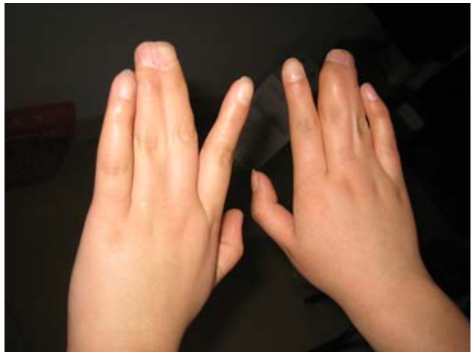

FA-IV22

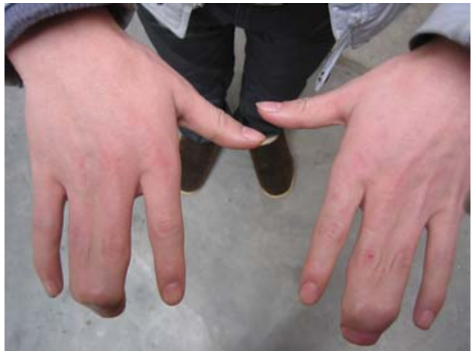

FA-IV23

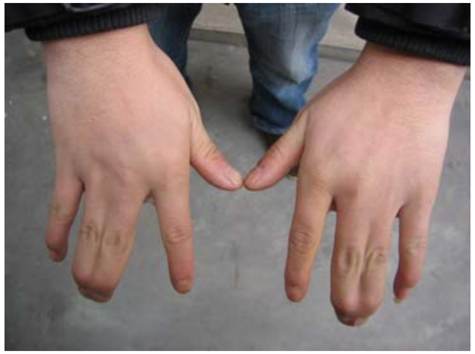

FA-IV25-surgery

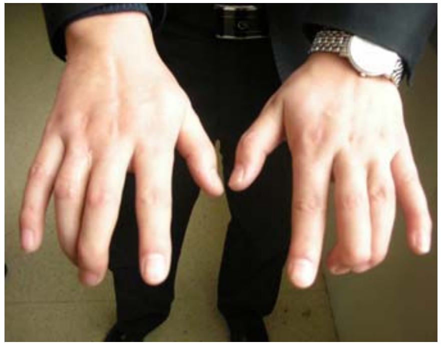

FA-IV26

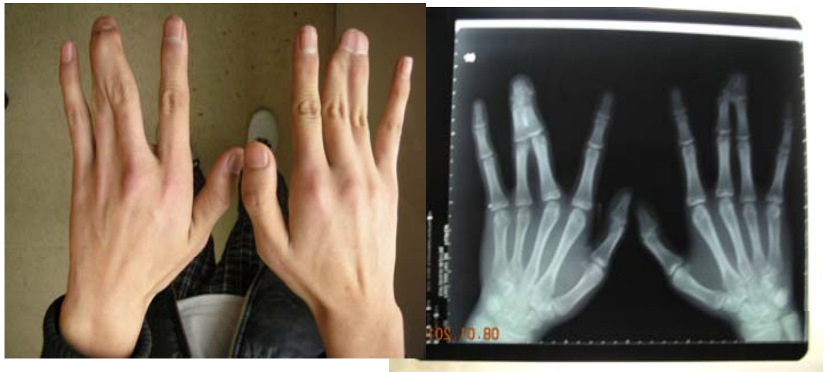

FA-V2-surgery

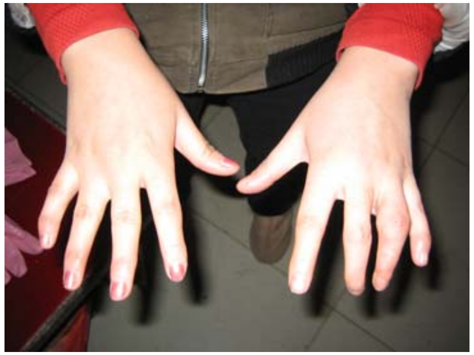

FA-V3

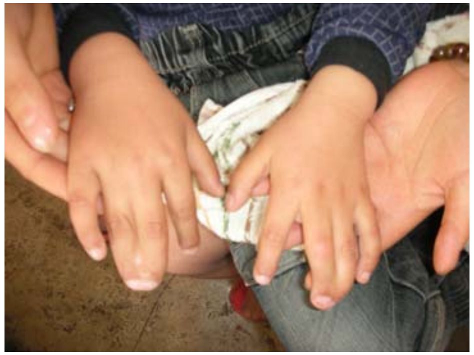

FA-V9

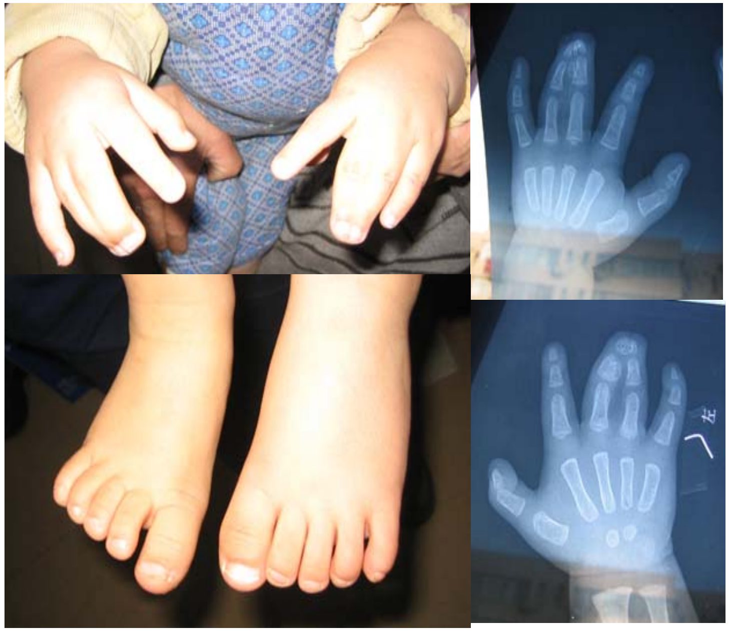

FA-V11-newborn

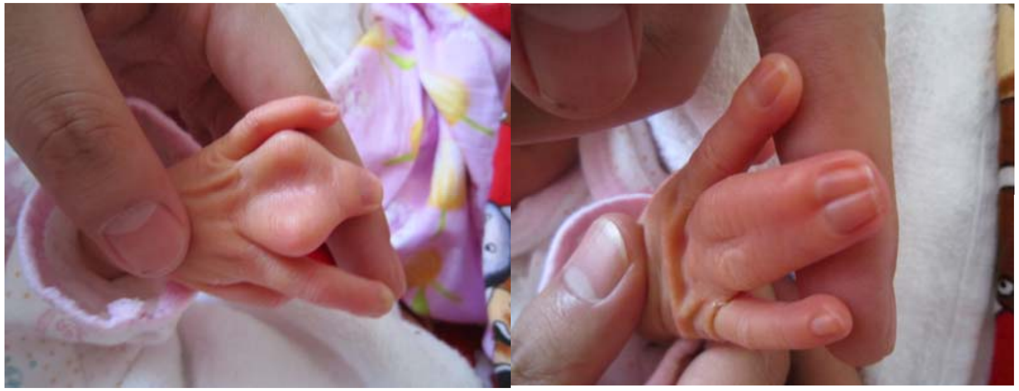

FB-I2

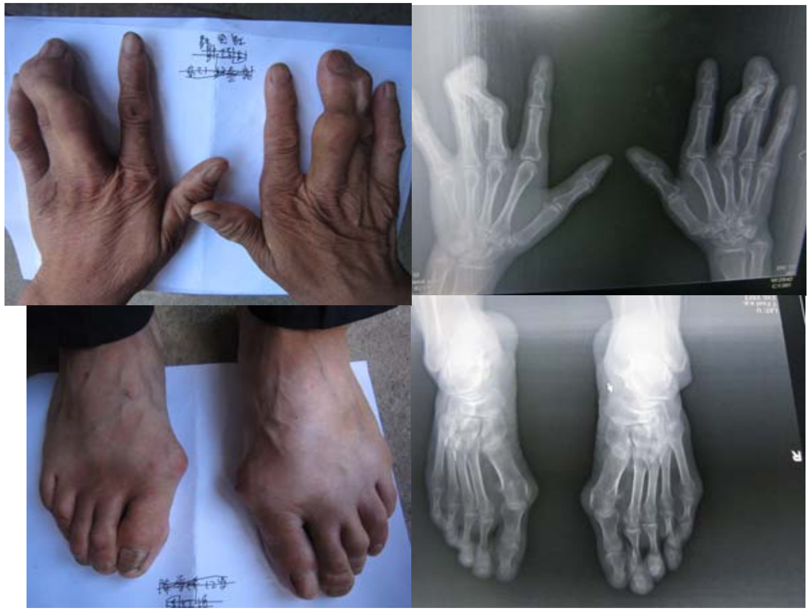

FB-III1--surgery

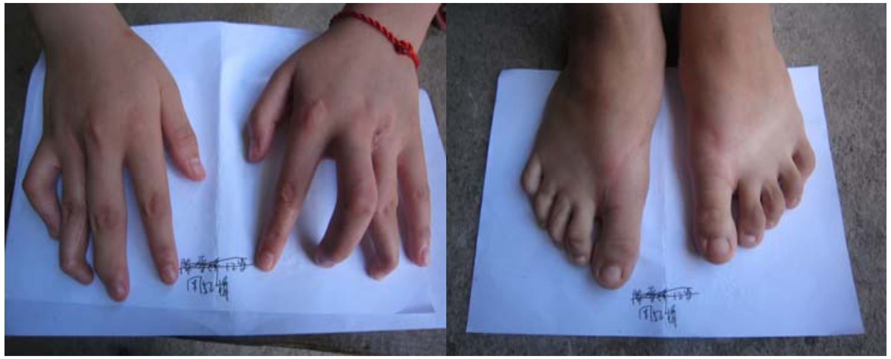

FB-III3—surgery

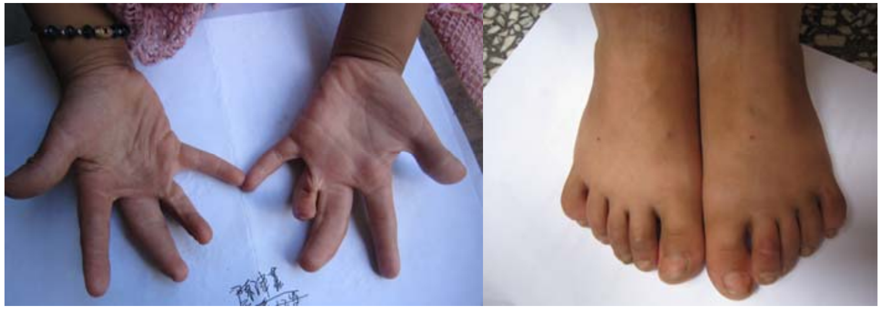

FB-III4-surgery

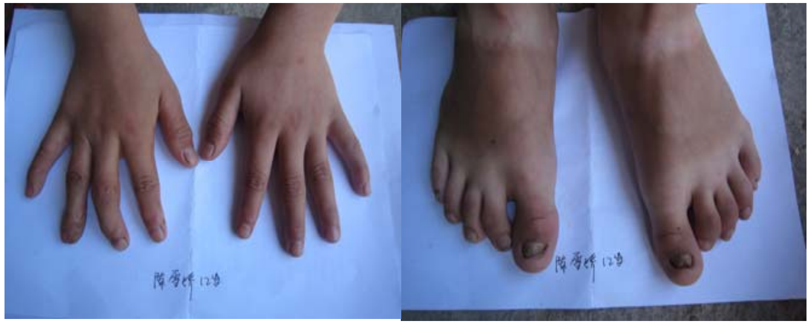

FB-III5-surgery

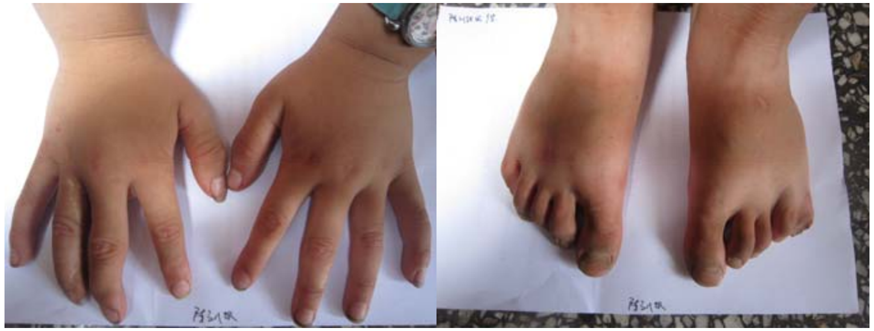

Supplement: Figure S1 — Photographs of affected individuals in family A and family B. (PDF) [file pone.0096192.s001.pdf]
